# LumbSten: The lumbar spinal stenosis outcome study

**DOI:** 10.1186/1471-2474-11-254

**Published:** 2010-11-02

**Authors:** Johann Steurer, Alexander Nydegger, Ulrike Held, Florian Brunner, Jürg Hodler, François Porchet, Kan Min, Anne F Mannion, Beat Michel

**Affiliations:** 1 Horten Centre for patient oriented research and knowledge transfer, University of Zurich, University Hospital, 8091 Zurich, Switzerland; 2 Department of Rheumatology and Institute for Physical Medicine of the University Hospital of Zurich, 8091 Zurich, Switzerland; 3 Department of Physical Medicine and Rheumatology, Balgrist University Hospital, Forchstrasse 340, 8008 Zürich, Switzerland; 4 Division of Diagnostic and Interventional Radiology of the University Hospital of Zurich, 8091 Zurich, Switzerland; 5 Spine Surgery Unit, Clinic Schulthess, Lengghalde 2, 8008 Zürich, Switzerland; 6 Spine Surgery Unit, Department of Orthopedics; Balgrist University Hospital, Forchstrasse 340, 8008 Zürich, Switzerland

## Abstract

**Background:**

Lumbar spinal stenosis is the most frequent reason for spinal surgery in elderly people. For patients with moderate or severe symptoms different conservative and surgical treatment modalities are recommended, but knowledge about the effectiveness, in particular of the conservative treatments, is scarce. There is some evidence that surgery improves outcome in about two thirds of the patients. The aims of this study are to derive and validate a prognostic prediction aid to estimate the probability of clinically relevant improvement after surgery and to gain more knowledge about the future course of patients treated by conservative treatment modalities.

**Methods/Design:**

This is a prospective, multi-centre cohort study within four hospitals of Zurich, Switzerland. We will enroll patients with neurogenic claudication and lumbar spinal stenosis verified by Computer Tomography or Magnetic Resonance Imaging. Participating in the study will have no influence on treatment modality. Clinical data, including relevant prognostic data, will be collected at baseline and the Swiss Spinal Stenosis Questionnaire will be used to quantify severity of symptoms, physical function characteristics, and patient's satisfaction after treatment (primary outcome). Data on outcome will be collected 6 weeks, and 6, 12, 24 and 36 months after inclusion in the study. Applying multivariable statistical methods, a prediction rule to estimate the course after surgery will be derived.

**Discussion:**

The ultimate goal of the study is to facilitate optimal, knowledge based and individualized treatment recommendations for patients with symptomatic lumbar spinal stenosis.

## Background

Pain radiating to lower extremities is a frequent complaint, especially in elderly people, and lumbar spinal stenosis is one of the underlying conditions. Lumbar spinal stenosis is defined as "buttock or lower extremity pain, which may occur with or without low back pain, associated with diminished space available for the neural and vascular elements in the lumbar spine"[1]. Narrowing can be localized at three different anatomic structures, the central canal, the lateral recess, or the neural foramina.

Patients complain of neurogenic claudication (pain in the buttocks and lower extremities with or without low back pain provoked by walking or extended standing and relieved by rest and bending forward) that is compatible with a narrowing of the lumbar spinal canal. In some patients Computer Tomography (CT) or Magnetic Resonance Imaging (MRI) can verify a stenosis in the lumbar spinal region, in others not. Vice versa a remarkable proportion of asymptomatic persons older 60 years show substantial narrowing of the spinal canal [2].

The incidence and prevalence of symptomatic lumbar stenosis are unknown. It is estimated from data in the USA that every year 90 out of 100.000 persons older than 60 years undergo lumbar surgery and lumbar spinal stenosis is the most frequent indication for this procedure [3,4]. In the Canton of Zurich with 1.3 Mio (2008) inhabitants we estimate that more than 300 operations on patients with lumbar spinal stenosis are performed every year.

### Treatment modalities

The natural course of spinal stenosis can vary, but is in most patients a relatively stable disorder, with severe disability and neurological deficits developing over time and not rapidly. In the SPORT trial it was reported that in most patients treated conservatively, symptoms did not worsen over four years [5]. A further trial focusing on development of pain over a five year period showed that in 70% of patients pain reached a plateau, in 15% pain increased over time and in 15% pain disappeared spontaneously [6,7].

For patients with moderate or severe symptoms different conservative and surgical treatment modalities are recommended, but knowledge about the effectiveness of these measures is scarce. Most of the studies evaluating non-operative treatments are of low quality and there is a lack of knowledge about the appropriate treatment of these patients [1,8]. There is little evidence that pharmacological treatment, including non-steroidal analgesics, calcitonin, methylcobalamin or intravenous lipoprostaglandin E, provides long-term benefit in patients with lumbar spinal stenosis [1]. A systematic review of the literature yielded insufficient evidence to draw conclusions regarding the effectiveness of physical therapy for lumbar spinal stenosis. In certain subgroups of patients physical therapy and exercise may be beneficial in controlling symptoms of neurogenic claudication in lumbar spinal stenosis, but the evidence that spinal manipulation offers benefit in the treatment is insufficient [1,8].

Epidural injections may have potential benefit and may be tried before surgery, but results about efficiency are mixed [9-13]. Some data suggest that epidural injection of corticosteroids relieves leg pain for a limited time but has no effect on the functional status or the need for surgery after one year [14]. The authors of a systematic review came to the conclusion that evidence is insufficient to recommend epidural injections in patients with spinal stenosis [15].

There is some evidence from randomized trials [5,16,17] and observational studies [18-22] that surgery improves symptoms in patients with spinal stenosis. The therapeutic effectiveness of conservative measures or surgery, as mentioned in the guidelines of the North American Spine Society, should be evaluated by further randomised controlled trials [1]. Such trials, however, as demonstrated in the study by Weinstein [16], are difficult to execute in patients with spinal stenosis. In the aforementioned study, patients with spinal stenosis were randomly allocated to either surgery or conservative treatment. Two years after randomization, only 67% of patients in the surgical group actually received surgery and 43% of those who were assigned to conservative treatment had also undergone surgery. Physicians and/or patients seem to have strong preferences for surgery or conservative therapy impeding the accomplishment of randomised trials [23].

The long term success rates of surgery vary between 45% and 72%, depending on the measured clinical outcome assessed (pain, walking capacity, neurologic symptoms, working ability) [24]. The outcome of surgery depends on a number of different factors. Indicators of postoperative outcome have been evaluated in different populations and the results have been summarized in a systematic review [25,26]. A lower self rated preoperative health-status, comorbidity, depression and limited, preoperative walking ability are strong predictors of an unfavorable clinical outcome.

## Methods/Design

### Study design

This multi-centre prospective cohort study includes patients with neurogenic claudication and radiological findings of lumbar spinal stenosis.

### Objectives

1. To develop a prognostic probability function (prediction rule) in order to predict the further course (one, two and three years after diagnosis) *in patients with lumbar spinal stenosis undergoing surgery.*

2. To develop (a) prognostic probability function(s) (prediction rules) in order to predict the further course (one, two and three years after diagnosis) in *patients with lumbar spinal stenosis treated, at least initially, by conservative measures.*

### Eligibility Criteria and identifying patients

Patients will be recruited in four hospitals in Zurich (University Hospital, University Clinic Balgrist, Schulthess Clinic and City Hospital Triemli). To be eligible for the study the participants must: 1) be aged 50 years or more; 2) have uni- or bilateral neurogenic claudication (defined by pain in the buttocks and/or lower extremities provoked by walking or extended standing and relieved by rest and/or bending forward; 3) have a verified diagnosis of spinal stenosis by Computer tomography or Magnetic Resonance Imaging (criteria are shown in table 1)[27]; 4) have an expected life expectancy of more than one year; 5) be able to give informed consent; 6) be available for follow-up and able to complete questionnaires in German language. Exclusion criteria are: 1) cauda equina syndrome requiring urgent surgery; 2) current fracture, infection or significant deformity (> 15° lumbar scoliosis); 3) current enrolment in another spine related treatment study; 4) clinically relevant peripheral arterial disease (confirmed by vascular specialist in patients without palpable pulses in the lower limb).

**Table 1:** Radiological criteria for lumbar spinal stenosis (L1 to L5)^!^

Location of stenosis	Radiological criteria (L1 to L5)
Central canal stenosis	Antero-posterior diameter of spinal canal ≤12 mm[31], or cross sectional area of dural tube ≤100 mm^2 ^[30]

Lateral recess stenosis	Lateral recess height ≤ 3 mm, or lateral recess depth ≤ 5 mm [32,34,35]

Foraminal stenosis	Foraminal diameter ≤ 5 mm [36]

^1^L lumbar vertebra

### Study sample size

Sample size is calculated for objective one, the development of a prognostic function for patients undergoing spine surgery, one year after surgery. Outcome after surgery will be quantified by the Swiss Spinal Stenosis Questionnaire (SSM)[28] and results will be dichotomized. An improvement of more than 0.5 points on the symptom severity scale and physical function scale in the SSM will be categorized as improved. According to the original publication [28] 0.5 points represents a clinically relevant improvement for the patients (Minimal Clinical Important Difference). We estimate that 60% of all patients with verified diagnosis will undergo surgery and two thirds of these (between 60% and 70%) of patients will show an improvement of more than 0.5 points in both scales (symptom severity and physical function) one year after surgery. To estimate the effect of ten prognostic variables we need roughly 100 patients without improvement, as defined above, after surgery. This leads us to include 350 patients in the study who undergo surgery.

### Procedure

**Figure 1:**
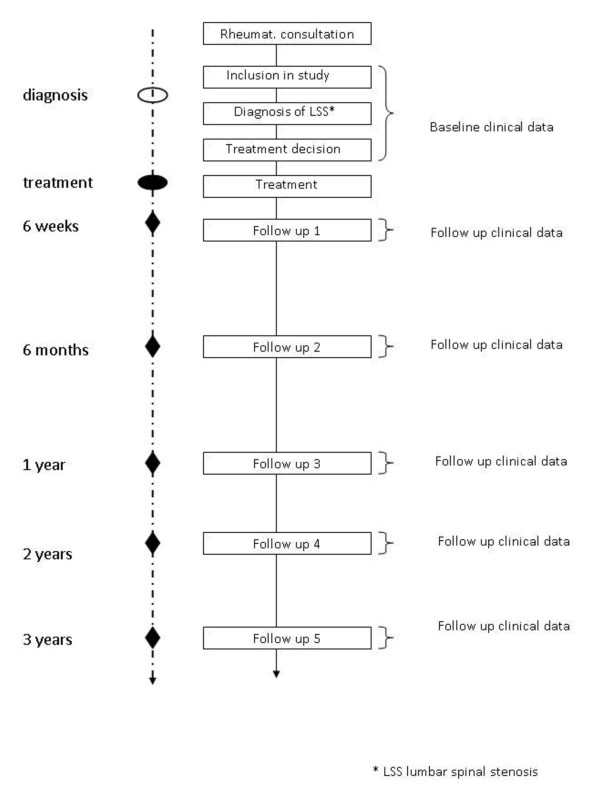
**Study flow**.

Patients will be recruited during consultations in the Rheumatology and Spine surgery units of all participating hospitals. Patients who fulfil the inclusion criteria will be informed about the goals and execution of the study and written informed consent will be obtained. Baseline and follow-up data will be collected by physicians assisted by study nurses. An overview of the study flow is given in Figure 1. In a preliminary phase of the study we will validate the German version of the Swiss Spinal Stenosis Questionnaire. This questionnaire is recommended for outcome measurement by the North American Spine Society.

After quality control all data will be stored in a central data base. All the recorded data and information contained within the study will remain strictly confidential. All the personal data will be made anonymous and assigned a code. The decoding will be stored at the Horten Centre and will be accessible only to the principle investigator. We will ensure that anonymization is maintained throughout any collaboration with other investigators. The data management will comply with the federal law of data protection of Switzerland.

### Treatment

The choice of treatment is not influenced by participation in the study and depends only on the clinical situation, physician's advice, and patients decision. Treatment options are watchful waiting, conservative treatment with physical therapy and/or drugs, epidural injections of analgesics and/or steroids and surgery. Detailed information about treatment(s) will be recorded using pre-specified questionnaires at each follow up visit. The questionnaire will comprise specific questions about: mode, duration and frequency of physical therapy; duration and dosage of drug treatments; site of epidural injection, with or without imaging, dosage of injected drugs; surgeon, precise and detailed description of surgical technique and procedure, intra- and postoperative complications, duration of surgical procedure and duration of hospital stay. Only experienced surgeons who have performed more than one hundred operations on the lumbar spine within the last two years will be included in the study.

### Data to be collected

At baseline information about socio-demographic characteristics, symptoms, clinical examination, consumption of analgesics and other treatments for lumbar spinal stenosis within the last 6 months will be collected. Radiological findings (CT, MRI) at different measurement points between lumbar vertebra 1 to lumbar vertebra 5 (central stenosis: antero-posterior diameter of spinal canal, cross sectional area of dural tube, interpedicular distance[29-31]; lateral stenosis: height and depth of the lateral recess [32-35]; foraminal stenosis: foraminal diameter will be quantified [36].

Comorbidities will be assessed with the validated Cumulative illness rating scale (CIRS) [37] which rates the presence and severity of comorbid diseases in 13 organ systems from 0 to 4, and sums organ-specific scores across systems to yield a summary score. Depression, a prognostic indicator, will be quantified with the Hospital Anxiety and Depression Score (HADS). The questionnaire consists of 14 questions, seven for anxiety and seven for depression [38].

Information about treatment will be collected at baseline for six months preceding enrolment in the study and at each follow-up visit for the period between the visits.

### Outcome measures (baseline and follow up)

Primary and secondary outcome data will be collected at baseline and at six weeks and six months after the time of treatment onset (surgery for the surgery group and time of enrolment for the nonsurgical group). Long-term outcome data will be gathered after one year and then annually up to three years.

### Primary Outcome

The Swiss Spinal Stenosis Questionnaire (SSM)[28] has been recommended by the North American Spine Society (NASS) [1] as the "gold standard" to quantify outcome in patients with spinal stenosis, will be used to quantify outcome. The SSM consists of three different subscales; severity of symptoms (possible range of the score is 1 to 5), physical function characteristics (possible range of scores is 1 to 4) and patient's satisfaction after treatment (the range of the scale is 1 to 4). In the present study, both the severity of symptoms and the physical function scales will represent the primary outcome, whereby a minimum of 0.5-point change in each will be required for the treatment to be considered a success. The SSM has been used as the primary outcome in different studies on lumbar spinal stenosis [39-43] and a German version is available.

### Secondary Outcomes

1. Bodily pain and physical function with 36-item Short-Form General Health Survey (SF-36) [44-46]. SF-36 scores range from 0 to 100, with higher scores indicating less severe symptoms.

2. Modified Oswestry Disability Index [46]. The Oswestry Disability Index ranges from 0 to 100, with lower scores indicating less severe symptoms.

### Ethics

The study will be conducted in accordance with the principles outlined by the 18^th ^World Medical Assembly, the Declaration of Helsinki (World Medical Association International Code of Medical Ethics (WMA) General Assembly, Pilanesberg, South Africa, October 2006) and all applicable amendments detailed by the World Medical Assemblies, the ICH (The International Conference on Harmonisation of Technical Requirements for Registration of Pharmaceuticals for Human Use) guidelines for Good Clinical Practice (GCP), and the Medical Research Involving Human Subjects Act of the Swiss Academy of Medical Sciences. This cohort study will be conducted in compliance with all international laws and regulations as well as any applicable guidelines.

### Statistical analysis

Epidemiologic data and patients' descriptive data available on continuous scales will be presented with medians, interquartile ranges or means and standard deviations as appropriate. Categorical data will be presented as rates and percentages. Associations of the continuous independent variables with the outcome variables will be reported using simple logistic regressions. Results from univariate analysis will guide multivariable modeling.

Assessment of associations will be performed using multi-variable models including potential confounders along with the independent variables of interest. Prognostic scores will be built using either logistic regression analysis or Cox proportional hazard models. Models will be validated in subsamples by cross validation. Calibration and discrimination of the cross-validated prognostic instruments will be assessed using the Brier Score.

## Discussion

This protocol describes the rational, methodology and design of a prospective, multi-center cohort study within hospitals in Zurich including patients with symptomatic lumbar spinal stenosis.

Based on the results of the study we will learn about indicators predicting the future course of patients with lumbar spinal stenosis with and without surgical therapy. These results will support physicians in informing patients, some of them suffering from more than one illness, about the expected course of the illness and help patients and physicians in deciding which therapy to choose.

## Competing interests

The authors declare that they have no competing interests.

## Authors' contributions

All authors participated in the study design; JS, AN and UH drafted the protocol. FB, JH, FP, AM, KM and BM critically reviewed the protocol and gave relevant input. All authors read and approved the manuscript.

## Pre-publication history

The pre-publication history for this paper can be accessed here:

http://www.biomedcentral.com/1471-2474/11/254/prepub
